# Stimulus-evoked phase-locked activity along the human auditory pathway strongly varies across individuals

**DOI:** 10.1038/s41598-020-80229-w

**Published:** 2021-01-08

**Authors:** Robin Gransier, Michael Hofmann, Astrid van Wieringen, Jan Wouters

**Affiliations:** Research Group Experimental Oto-rhino-laryngology (ExpORL), Department of Neurosciences, KU Leuven, Herestraat 49, Box 721, 3000 Leuven, Belgium

**Keywords:** Neuroscience, Auditory system, Cortex

## Abstract

Phase-locking to the temporal envelope of speech is associated with envelope processing and speech perception. The phase-locked activity of the auditory pathway, across modulation frequencies, is generally assessed at group level and shows a decrease in response magnitude with increasing modulation frequency. With the exception of increased activity around 40 and 80 to 100 Hz. Furthermore, little is known about the phase-locked response patterns to modulation frequencies ≤ 20 Hz, which are modulations predominately present in the speech envelope. In the present study we assess the temporal modulation transfer function (TMTF_ASSR_) of the phase-locked activity of the auditory pathway, from 0.5 to 100 Hz at a high-resolution and by means of auditory steady-state responses. Although the group-averaged TMTF_ASSR_ corresponds well with those reported in the literature, the individual TMTF_ASSR_ shows a remarkable intersubject variability. This intersubject variability is especially present for ASSRs that originate from the cortex and are evoked with modulation frequencies ≤ 20 Hz. Moreover, we found that these cortical phase-locked activity patterns are robust over time. These results show the importance of the individual TMTF_ASSR_ when assessing phase-locked activity to envelope fluctuations, which can potentially be used as a marker for auditory processing.

## Introduction

Human speech is characterized by a rhythmic stream of amplitude and frequency fluctuations that convey phoneme, syllable, word, and phrase information^1^. The ability of the auditory system to process these fluctuations is of importance for speech perception. Envelope modulations in particular, have been shown to be essential for speech perception^2–4^. The human auditory system is capable of processing highly degraded speech as long as the temporal envelope modulations below 20 Hz are preserved^2–5^. Nevertheless, envelope modulations up to 200 Hz contribute to speech understanding, especially in adverse listening situations^6,7^.

The neural ensembles of the auditory system must be able to encode these envelope fluctuations in order to perceive speech intelligibly. In its simplest form these envelope fluctuations can be represented as single-frequency amplitude modulations (AM). AM perception and the responsiveness of the auditory pathway to AM sounds have been investigated in numerous behavioral and neurophysiological studies. AM perception is behaviorally often assessed by means of the minimum perceivable modulation depth needed for AM detection^8^. The temporal modulation transfer function (TMTF), which reflects the minimum perceivable modulation depth as a function of modulation frequency, has a general low-pass characteristic. It is relatively constant up to 50 Hz and then declines with 4–5 dB per octave^9^.

This low-pass characteristic is also observed in the responsiveness of the ascending auditory pathway. In vivo studies in animals have shown that earlier neural regions in the ascending auditory pathway are responsive to higher modulation frequencies whereas cortical regions predominately respond to lower modulation frequencies^10^. fMRI studies in humans have shown that although all neural regions across the ascending human auditory pathway are responsive to AM sounds ranging from 4 to 256 Hz, subcortical regions have a greater activation to modulation frequencies > 32 Hz, whereas cortical regions have more activation to modulation frequencies < 32 Hz^11,12^.

Phase-locking to the temporal envelope is considered as an important mechanism for AM processing^10^ and is often assessed in humans with the auditory steady-state response (ASSR)^13^. ASSRs are electrophysiological responses that reflect the phase-locking ability of neural ensembles in the auditory pathway. They are typically evoked by repetitive varying acoustic signals, such as click trains or amplitude modulated (AM) sounds^13,14^. ASSRs to modulation frequencies within 80–100 Hz originate predominantly from the brain stem structures^15–18^. Reducing the modulation rate results in a shift of the phase-locked activity towards the higher regions of the auditory pathway. The 40-Hz ASSR, which is the first and one of the most reported ASSRs^14^, originates predominantly from the left and right auditory cortex^16–19^, but also has generators in the thalamus^17,18^, and brain stem^16–18^. Modulation frequencies ≤ 20 Hz predominantly originate from the auditory cortex^16–18^.

Ample studies in the literature investigate the relation between differences in response strength across subjects to various modulation frequencies and speech perception^20–27^, modulation detection^28,29^, gap detection^28^, and loudness perception^30,31^. In addition, differences in ASSR strength across subjects are also used to study, for example differences in temporal processing in dyslexia^32–34^, cochlear implant users^27,29,35^, aging^36^ and schizophrenia^37^. These associations are, however, often assessed with ASSRs to a distinct number of modulation frequencies. Furthermore, the temporal modulation transfer function of the ASSR (TMTF_ASSR_), which reflects the magnitude of the ASSR, measured at the scalp in response to a wide range of modulation frequencies, is often not taken into account. We, however, argue that knowledge about the phase-locking ability of the auditory pathway to different modulation frequencies, as assessed with ASSRs, and its intersubject variability is of importance when investigating the association between phase-locked activity and functional outcomes that rely on temporal processing.

The TMTF_ASSR_ has only been reported in a few studies. Purcell et al.^28^ and Poulsen et al.^38^ assessed the TMTF_ASSR_ for modulation frequencies ranging from 20 to 600 Hz and found that the TMTF_ASSR_ peaks between 30 and 60 Hz and around 80–100 Hz. Similar results have been reported by Ross et al.^39^. In addition, Poulsen et al.^38^ found that the peak in ASSR strength within the 30–60 Hz range shifts with increasing age. Both studies give insight in the general TMTF_ASSR_ in humans, but not directly in the inter-subject variability of the TMTF_ASSR_. Furthermore, only a limited amount of studies^20,40,41^ investigated the TMTF_ASSR_, based on a limited selection of modulation frequencies, for envelope fluctuations that are present in the speech envelope and which originate from the auditory cortex (i.e. modulation frequencies ≤ 20 Hz).

We hypothesize that intrinsic differences in the individual phase-locked activity to different modulation frequencies along the auditory pathway exist and that these are reflected in the individual TMTF_ASSR_. These differences in phase-locking ability are potentially related to temporal auditory processing and could provide valuable insight in auditory processing in general. In the present study we investigated the phase-locked activity along the auditory pathway at a high resolution (i.e. the TMTF_ASSR_ is characterized based on 70 independent measurements/modulation frequencies) to AM sounds with modulation frequencies between 0.5 and 100 Hz in a homogeneous group (i.e. hearing status, age, and cognitive abilities) of normal-hearing adults.

## Methods

### Participants

Twenty-five normal-hearing young adults participated in the EEG experiments (mean age = 22.2 years, SD = 1.9, male = 3). All subjects had normal hearing. Hearing thresholds for both ears were ≤ 20 dB for the octave frequencies ranging from 250 to 4000 Hz. All subjects were right handed, except for S8 who was left handed and S25 who was ambidextrous. The Medical Ethics Committee of the UZ Leuven approved this study (Approval number: B322201214866) and all methods were carried out in accordance with the relevant guidelines and regulations. Written informed consent was obtained from all participants before testing.

### Stimuli

A 100% sinusoidally amplitude modulated, one-octave band white noise, centered at 1 kHz was used as stimulus. Seventy stimuli, each with a different modulation frequency, were used to characterize the TMTF_ASSR_ within the 0.5–100 Hz range. The step size between adjacent modulation frequencies depended on the modulation frequency. The step size was 0.5, 1, and 2 Hz for the range 0.5–10, 10–20, and 20–100 Hz, respectively. Non-integer modulation frequencies had an epoch length of 2.048 s, whereas integer modulation frequencies had an epoch length of 1.024 s. Each stimulus had a duration of 5.12 min (i.e. 150 or 300 epochs of 2.048 or 1.024 s, respectively), and to ensure that no phase drift occurred across epochs, modulation frequency values were adjusted so that each epoch contained an integer number of cycles (In the following only the rounded to one decimal modulation frequencies are reported). All stimuli were presented to the left ear and at a sound pressure level of 70 dB re 20 µPa.

Stimuli were generated on a laptop with custom-written software^42^ interfacing with an external sound card (RME—Hammerfall DSP Multiface II) through a FireWire connection. An insert earphone (3 M E-A-RTONE™—3A), housed in an electrically grounded casing (Perancea—CFL2), was used to present the stimuli to the subject. A trigger was sent at the start of each epoch.

Calibration of the sound emitting system and stimuli was done with a sound level meter [Brüel & Kjær (B&K)—2250] that was connected to a half-inch microphone (B&K—4189) which was configured in a 2-cc coupler artificial ear (B&K—4152).

### EEG recordings

A 64-channel EEG system (Biosemi—ActiveTwo) was used to record the EEG during the measurements. A sample rate of 8192 Hz was used and the system had a built-in low-pass filter with a cutoff frequency of 1638 Hz. All recording electrodes were placed on the participant's head by means of a cap and according to the international standardized 10–20 system (Jasper 1958). Recordings were made in a sound booth which was electrically shielded. The ambient noise within the sound booth was conform the ANSI 3.1 standard^43^. Participants watched a silent movie with subtitles and sat in a comfortable chair during the recordings and the presentation of the stimuli. This was done to ensure an attentional state as similar as possible across subjects and recording moments. Subjects were asked to move as little as possible during the presentation of the stimuli to reduce the effect of movement artifacts on the EEG-recordings. This approach was identical as that used in Gransier et al.^44^.

Subjects attended two to three sessions for the total recording of the TMTF_ASSR_. Each session including breaks lasted between ~ 3 and 4 h and the total effective recording time to obtain the whole TMTF_ASSR_ was 6 h. The recordings of the ASSRs to the different modulation frequencies were done in ascending order.

We measured the TMTF_ASSR_ to modulation frequencies between 0.5 and 20 Hz a second time in ten subjects (i.e., S01, S03, S04, S14, S20, S21, S22, S23, S24, and S25) to assess if the cortical obtained TMTF_ASSR_ were not affected by attention or state of arousal. During the retest session the modulation frequencies were presented in random order. The time between the test and retest session was on average 59 days, (SD = 38.8 days, range = 7–114 days). To assess the robustness of the TMTF_ASSR_ between 0.5 and 20 Hz we compared the absolute difference between the ASSR amplitudes, per modulation frequency, and subject with the neural background noise. The ASSR amplitude, measured at the scalp, is assumed to be stable over time and is the combination of the phase-locked activity (the true ASSR) and the non-phase-locked neural background noise. The neural background noise determines the within measurement variability, which is expressed as the standard deviation of the mean^44^. Robustness of the response ($${t}_{ASSR})$$ was assessed based on the difference of each ASSR with the mean across the two sessions divided by average neural background noise across the two sessions (see Eq. 1)

1 $${t}_{ASSR\left[S,fmod\right]}= \frac{\left|{A}_{ave\left[S, fmod\right]}-{A}_{test|retest[S, fmod]}\right|}{{\sigma }_{ave\left[s, fmod\right]}}$$

where $S$ is the subject and $fmod$ the modulation frequency used to evoke the response. ${A}_{test}$ and ${A}_{retest}$ are the amplitudes of the ASSR to $fmod$ for the test and retest sessions, respectively. ${A}_{ave}$ is the power based average amplitude of the two measurements and ${\sigma }_{ave}$ is the power based average non-phase-locked activity amplitude of the two measurements divided by $\sqrt{2}$. A good reproducibility is obtained, taking a normal distribution into account, when ≥ 68% and ≥ 95% of the absolute differences is within 1 and 1.96 times ${\sigma }_{ave}$, respectively.

### Signal processing

**Figure 1:**
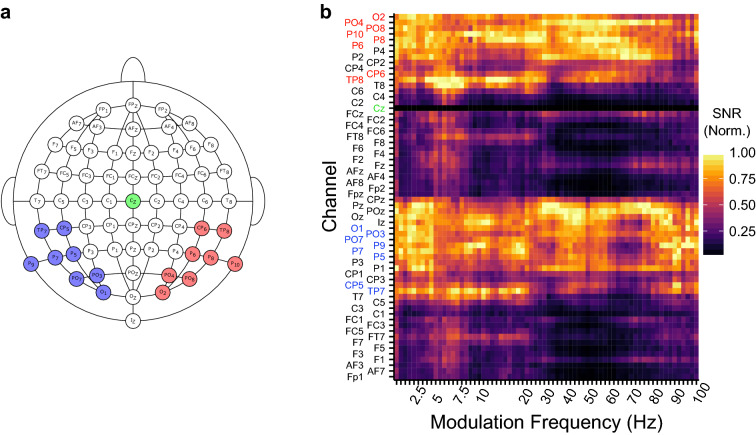
Electrode configuration and response strength across the scalp. (**a**) The electrodes that were combined into the left (in blue) and right (in red) hemispheric channel, and the reference electrode Cz (in green). (**b**) The average SNR (linear) normalized to the maximum value per modulation frequency in function of modulation frequency and recording channel. A SNR (Norm.) of 1 represents the recording electrodes with the highest SNR for a particular modulation frequency whereas a 0 indicates the recording electrodes with the lowest SNR. The results in panel B show that the recording electrodes that were used in the analyses were those with the highest SNR across modulation frequencies.

Signal processing was done in Matlab^45^ (version R2013A). Time signals of the recording electrodes located within the parietal-temporal and occipital regions were averaged into a left, and a right hemispheric recording channel. Recording electrodes TP7, CP5, P9, P5, P7, PO7, PO3, and O1 formed the left hemispheric recording channel, whereas TP8, CP6, P10, P6, P8, PO8, PO4, and O2 formed the right hemispheric recording channel (Fig. 1), except for the repeatability measures where both channels were averaged together [i.e. to obtain the best signal-to-noise-ratio (SNR)]. These electrodes were chosen to allow a comparison across hemispheres, and because the largest SNRs could be obtained from these hemispheric specific regions across the different modulation frequencies (Fig. 1b). Recording electrodes and channels were high-pass filtered with a 2nd order Butterworth high-pass filter with a cutoff frequency of 2 Hz to remove any DC component in the recordings. After filtering, each time signal of each electrode and recording channel was, based on the triggers divided into individual epochs with a length of 1.024 or 2.048 s. 5% of the epochs with the highest peak-to-peak amplitude were removed from the recordings as they were assumed to contain muscle and other recording artifacts.

A Fast Fourier Transform was used to calculate the complex frequency spectrum of each of the remaining epochs, resulting in a frequency resolution of 0.49 or 0.98 Hz. The different channels and electrodes were referenced to the Cz electrode by subtracting the complex frequency spectrum of the reference electrode from the complex frequency spectrum of each channel. To compensate for the filter effects on the magnitude of the response, the inverse gain of the high-pass filter was applied to the frequency spectrum of each epoch. For each epoch the biased response power, amplitudes, and phases were obtained from the complex frequency spectrum corresponding to the modulation frequencies as used during the experiment (i.e., the response spectrum). Mean biased response amplitudes and phases were computed by vector averaging the complex response spectrum across epochs. The biased response amplitudes—i.e. the average response amplitude directly derived from the EEG recordings—were used as the noise-corrected response amplitudes^46^—i.e. the response amplitudes from which the noise is subtracted (power based)—would unjustifiably affect the ASSRs evoked with the lower modulation frequencies due to the 1/f noise pattern inherent to EEG activity. The neural background noise was based on the ‘standard deviation of the mean’ which is calculated as the standard deviation over epochs divided by the square-root of the number of epochs^44^.

Biased SNR was calculated by dividing the mean biased response power (i.e. signal + noise power) by the power of the neural background activity, and was then converted to a dB value. A one-sample Hotelling T^2^^47–49^ was used, for each channel, to determine if the synchronized activity (i.e., the measured response) differed significantly from the nonsynchronized neural background activity. A significance level of 5% was applied.

### Data and statistical analysis

Given the large amount of modulation frequencies tested within this study we were able to determine the latencies based on the apparent latency^13,50^. Apparent latencies were determined based on the phase delay across modulation frequencies. We used the unwrap function in R to compute the phase delay across modulation frequencies, i.e. to transpose the phase in a circular demission to a continuum, for each subject individually. Given that the TMFT_ASSR_ was measured with very small frequency steps ensured that no erroneous unwrapping occurred. The apparent latencies were then calculated with a moving frequency window of 10 Hz and with a step size of 5 Hz. The apparent latency was calculated, for each participant and channel, within each moving frequency window by dividing the absolute slope of the phase delay across the significant response frequencies within each moving frequency window by 360°^48,50^. A criterion of four significant responses within a frequency window was used to calculate the apparent latency.

Hemispheric laterality was assessed by the laterality index (EQ. 2). Where A_right_ and A_left_ are the biased response amplitudes of the right and left hemispheric channel, respectively. The LI was only calculated when the responses of the left and right hemispheric channel met one of the following two criteria: (1) both channels have significant responses, or (2) in the case only one channel has a significant ASSR, the channel with the significant response needs to have a SNR > 6 dB and the absolute difference between the neural background noise of both channels needs to be equal or less than 28.3 nV. The cutoff of 28.3 nV is based on the average plus one standard deviation of the absolute difference between the neural background noise of the both hemispheres across all subjects and modulation frequencies. These criteria prevented false LIs to be calculated based on the difference between the noise levels across hemispheres. 77.86% of the data met the inclusion criteria.

2 $$LI= \frac{{A}_{right}-{A}_{left}}{{A}_{right}+{A}_{left}}$$

Although many studies in the literature refer to 40 Hz as the peak of maximum activity within the 30–60 Hz region, there is only limited amount of research that shows that 40 Hz is indeed the modulation frequency that evokes the largest ASSR^38,39^. To gain insight in the maximum peak of activity we therefore calculated the peak frequency (f_peak_) as the weighted sum of the spectral estimates divided by the total power within the range^51^ (EQ. 3)

3 $${f}_{peak}= \frac{\sum_{i=1}^{n}{fmod}_{i}\cdot {A}_{i}^{2}}{\sum_{i=1}^{n}{A}_{i}^{2}}$$

where n is the number of modulation frequencies used to evoke ASSRs within the 30–60 Hz range, *f*mod_i_ the modulation frequency and *A*_i_^2^ the power of the ASSR evoked with *f*mod_i_.

Statistical analysis was carried out in R^52^ (version 3.4.3). Parametric tests were used in case the data included in the analysis met the assumptions for parametric testing. Otherwise, non-parametric tests were used. To assess the relationship between the ASSR strength evoked with the different modulation frequencies we computed the Pearson correlation coefficient between the amplitudes of each combination of modulation frequencies (e.g. 40 Hz vs 42 Hz). Each separate correlation analysis encompasses all subjects who were assessed with both modulation frequencies. This resulted in a minimum of 21 and a maximum of 25 data points included in each analysis. In all statistical analyses a significance level of 5% was used and was adjusted to 1% in case of multiple comparisons.

## Results

In total 1721 ASSR recordings (modulation frequencies × subjects) were conducted, excluding the retest measures. Corresponding to 146.8 h of EEG data. Due to time constrains and/or measurement errors only 29 recordings, of the intended 1750 measurements (i.e. 1.7%) across all subjects, were absent or could not be used in the analyses. The modulation frequency with the lowest number of subjects included was 96 Hz (N = 21).

### Percentage significant responses

**Figure 2:**
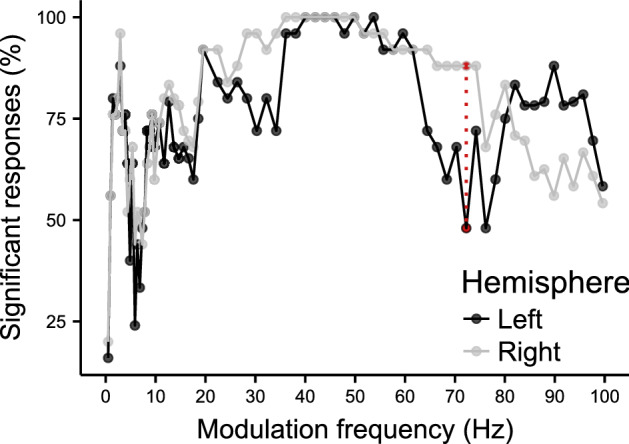
Percentage of significant responses as a function of modulation frequency. The red dashed line indicates the modulation frequency that resulted in a significant difference in the number of significant responses between the left and right hemispheric channel (*p* = 0.006).

Significant ASSRs could be evoked in all subjects within the 40–50 Hz region. Modulation frequencies that resulted in significant responses in less than 50% of the subjects, when taking both hemispheres into account, were 0.5, 5, 6, 6.5, 7.5, 72, and 76 Hz (Fig. 2). We observed a difference in the number of significant ASSRs between the left and right hemispheric channel mainly within the 60–100 Hz region, this difference was only significant at 72 Hz (χ^2^(1), p = 0.006).

### Response strength

#### Group average results

**Figure 3:**
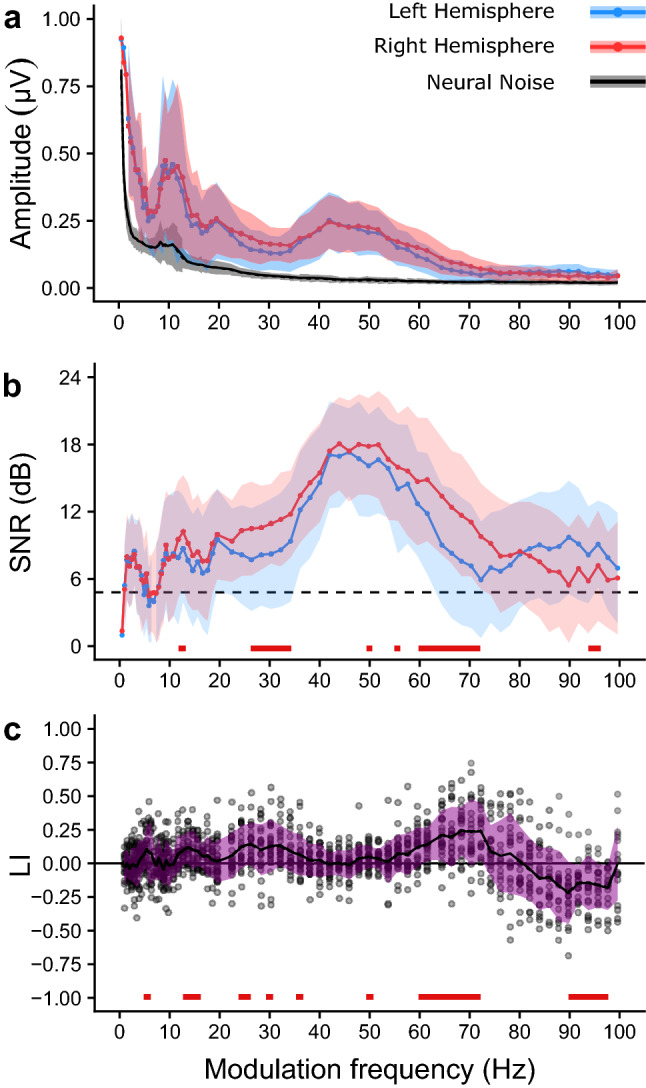
The group average biased (**a**) amplitude, (**b**) signal-to-noise ratio (SNR), and (**c**) laterality index as a function of modulation frequency. The mean is represented by the solid line and the shaded area reflects the range between the mean ± one standard deviation. The solid black line and the gray area in panel A show the average neural background noise across channels ± one standard deviation, respectively. The individual LIs are represented by the dots in (**c**).

The ASSR strength was characterized in terms of biased amplitude and response SNR. The amplitude group averaged TMTF_ASSR_ showed a typical low-pass pattern with the exception of peaks at 9–12 Hz (mean_across channels_ = 441 nV, SD_across channels_: 275 nV), 20 Hz (mean_across channels_ = 254 nV, SD_across channels_ = 134 nV) and 40–52 Hz (mean_across channels_ = 226 nV, SD_across channels_ = 94 nV) (Fig. 3a). When taking the SNR of the ASSR into account, we observed the highest SNR for ASSRs evoked within the 40–52 Hz region (mean_across channels_ = 17 dB, SD_across channels_ = 4.6 dB) (Fig. 3b).

The effect of modulation frequency was assessed based on the SNR. By doing so the magnitude of the 1/f neural background noise did not have a direct effect on the statistical analysis. There was a significant effect of modulation frequency on the SNR for both the left (*H*(69) = 564.78, p < 0.001) and right hemispheric channel (H(69) = 724.35, p < 0.001). We conducted pairwise Wilcoxon signed rank test to gain insight in the effect of the hemispheric channel on the obtained SNR per modulation frequency. SNRs as derived from the right hemispheric channel were significantly higher than the SNRs as derived from the left hemispheric channel (p < 0.01) in the regions 12–13 Hz, 26–34 Hz, 50–72 Hz with the exception of 52, 54 and 58 Hz, and between 94 and 96 Hz.

The laterality index was used to gain more insight in the effect of hemispheric lateralization of the ASSR as a function of modulation frequency (Fig. 3c). A one-sampled *t* test, corrected for multiple comparisons, was used to test if the obtained LI values were significantly different from 0 (i.e. no laterality). There was a significant right lateralization for ASSRs evoked with the modulation frequencies 5.5, 13–16, 24–26, 30, 36, 50, and 60–72 Hz (p < 0.01), whereas there was a significant left lateralization for ASSRs evoked within the 90–98 Hz range. The highest absolute LI (0.75) was obtained in S25 at 70 Hz, the corresponding ASSR amplitudes were 13 nV (noise: 13 nV) and 92 nV (noise: 16 nV) for the left and right hemispheric channel, respectively.

#### Correlation between response amplitudes across modulation frequencies

**Figure 4:**
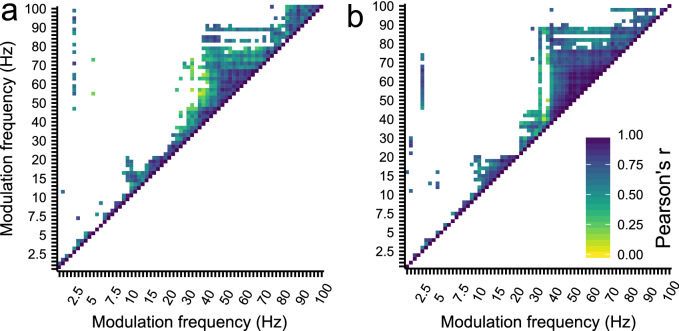
The correlation matrix of the biased response amplitudes across modulation frequencies for (**a**) the left and (**b**) the right hemispheric channel. Only p < 0.01 and r > 0 is shown. Four distinct patterns are apparent from the correlation matrixes, namely the correlation between 2.5 Hz and ~ 50–100 Hz, between 8 and 10 Hz and 10–20 Hz, the correlation between ASSRs within the 40–90 Hz region, and finally the correlation between ASSRs within the 80–100 Hz region. These patterns indicated that the response magnitude to a specific modulation frequency are associated with the response magnitude of the ASSRs that fall within a specific cluster.

**Figure 5:**
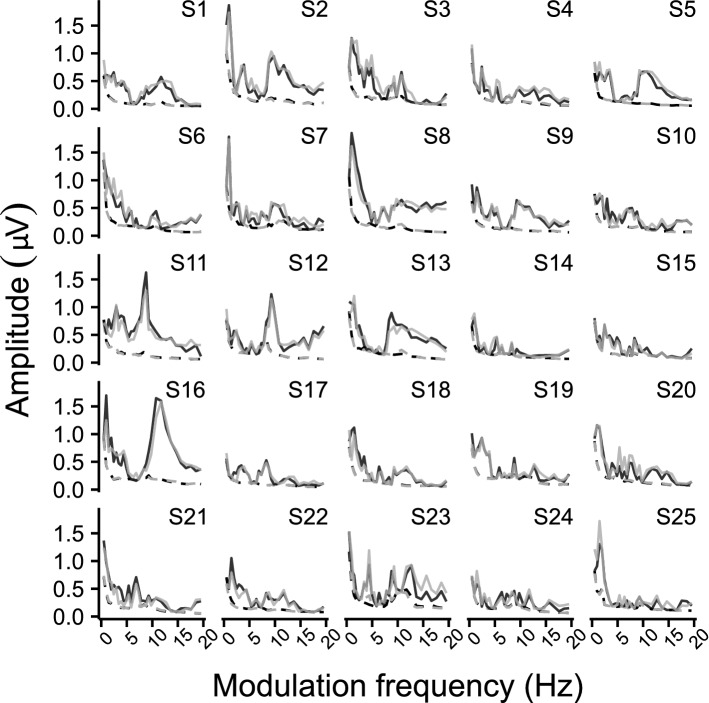
Individual TMTF_ASSR_ within the 0.5–20 Hz region. The different panels show the biased response amplitude as a function of modulation frequency per subject. Solid lines represent the ASSR amplitude, whereas the dashed lines represent the neural background noise. The left and right hemispheric channel are shown in black and gray, respectively. Comparing the subject-specific TMTF_ASSR_ show that there is a high inter-subject variability in response magnitude for ASSRs evoked with modulation frequencies ≤ 20 Hz.

**Figure 6:**
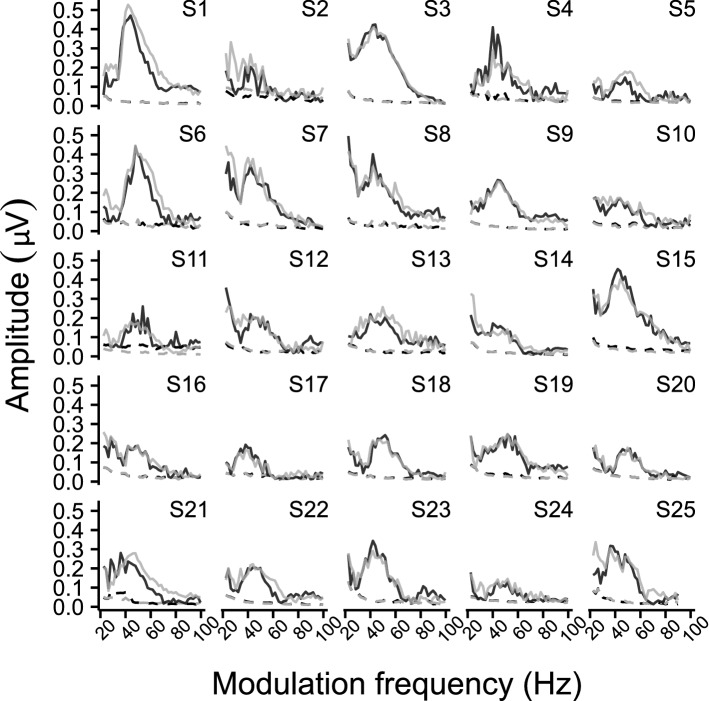
Individual TMTF_ASSR_ within the 20–100 Hz region. The different panels show the biased response amplitude as a function of modulation frequency per subject. Solid lines represent the ASSR amplitude, whereas the dashed lines represent the neural background noise. The left and right hemispheric channel are shown in black and gray, respectively. Comparing the subject-specific TMTF_ASSR_ shows that all subjects had an increased ASSR magnitude, although differences exist across subjects, for ASSRs evoked with modulation frequencies ranging from 30 to 60 Hz and 80–100 Hz.

Different generators can contribute to the obtained ASSR at scalp level and the responsiveness of each generator to different modulation frequencies may vary. In addition, constructive interference between the dipoles of the different generators can affect the measured scalp potentials^53^. Correlation analysis of the ASSR amplitudes across modulation frequencies was used to gain insight in the patterns of similar activity across the generators that contribute to the obtained scalp recorded ASSR. We computed a correlation matrix, using the Pearson’s correlation coefficient (p < 0.01), to gain insight in the overlap in overall responsiveness of the generators that contribute to the scalp recorded ASSR across modulation frequencies, for both the left and right hemispheric channel (Fig. 4). Four distinct patterns are apparent from the correlation analysis. First, the ASSRs evoked with 2.5 Hz is correlated with ASSRs evoked with modulation frequencies within the ~ 50–100 Hz, and 50–75 Hz for the left and right hemispheric recording channel, respectively. Second, there is a correlation between the ASSRs evoked with 8–10 Hz and 10–20 Hz. Third, there is a correlation between the ASSRs evoked within the 40–90 Hz range, and finally, there is a correlation between the ASSRs evoked within the 80–100 Hz region, which is more distinct for the ASSRs obtained from the left hemispheric recording channel. These results suggest that at a group level the response magnitude to a specific modulation frequency within a cluster is representative of the relative position of the response strength of a specific subject within the group compared to the other modulation frequencies within that cluster. This is also apparent from the individual TMTFs_ASSR_ as shown in Figs. 5 and 6.

### Intersubject variability in TMTF_ASSR_

**Figure 7:**
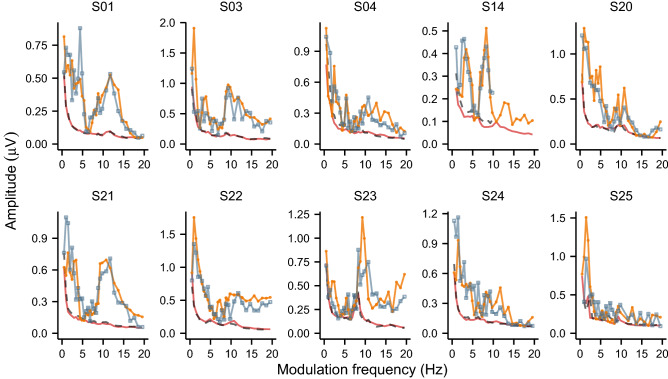
The TMTF_ASSR_ within the 0.5–20 Hz region obtained during the test and retest sessions (N = 10). Each panel show the individual phase-locked activity averaged across the left and right hemispheric channel for the test (orange) and retest (blue) session, and the neural background noise for the test (red) and retest (black) session. The retest data of S14 was only available for 0.5–10 Hz. The data show that the TMTF_ASSR_ was robust across sessions, indicating that these phase-locked activity patterns are inherent to each subject.

Given the large amount of data and the long recording durations per modulation frequency it was possible to gain a detailed insight in the intersubject variability of the TMTF_ASSR_. We found a large intersubject variability in the responsiveness of the auditory pathway to modulation frequencies within the 0.5–20 Hz range. These distinct response patterns originate predominately from cortical generators (see “Phase delay and apparent latencies”). Moreover, we assessed if these subject-unique and modulation-frequency dependent response patterns were not a consequence of attention or state of arousal by measuring the ASSRs within the 0.5–20 Hz range on a second occasion in ten subjects. The period between the test and retest session was on average 59 days (SD = 38.8 days, range 7–114 days). It is apparent from Fig. 7 that the measured patterns are indeed subject-dependent and similar over time. The absolute amplitude difference between the test and retest session was within 1 and 1.96 times the average neural background noise (i.e. the within measurement variability) of a single measurement in 81.6% and 97.5% of the cases, thereby indicating a good reproducibility of the evoked ASSRs.

**Figure 8:**
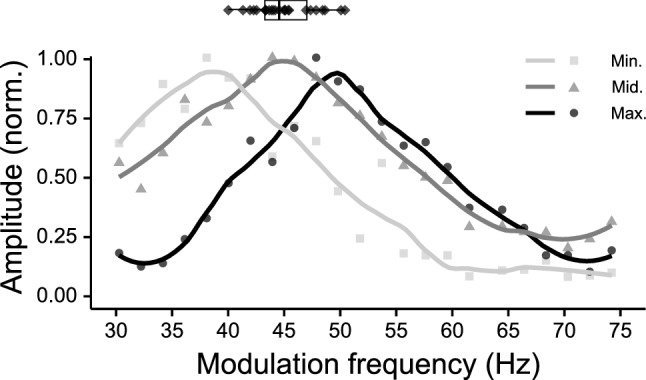
The individual f_peaks_ within the 30–60 Hz range are represented in black diamonds and its distribution as a boxplot. The three curves show the responsiveness of three different subjects that are representative for the intersubject variability of the response patterns from which the peak frequencies are derived (i.e. min, mid, and max are from the subject with the lowest, around the median, and the max peak frequency within the group, respectively). All curves are normalized to the maximum value of each subject and are smoothed for illustrative purposes.

In contrast to the TMTF_ASSR_ within the 0.5–20 Hz range, similar overall response patterns were obtained for modulation frequencies within the 20–100 Hz range (Fig. 6). Nevertheless, there is also a large subject variability in the responses obtained in this range. First, the maximum response amplitude within this range differed considerably across subjects (mean = 314 nV, SD = 107 nV, range 179–527 nV). Second, we found a large variation in modulation frequency (f_peak_) that resulted in the largest response amplitude within the 30–60 Hz range (Fig. 8). There was no significant difference between the f_peak_ as obtained with the left and right hemispheric channel (*t*_paired_(24) = − 0.106, p = 0.92). The f_peak_ varied across subject between 40.0 and 50.4 Hz and was, on average, 45.0 Hz (SD: 2.7 Hz).

### Phase delay and apparent latencies

**Figure 9:**
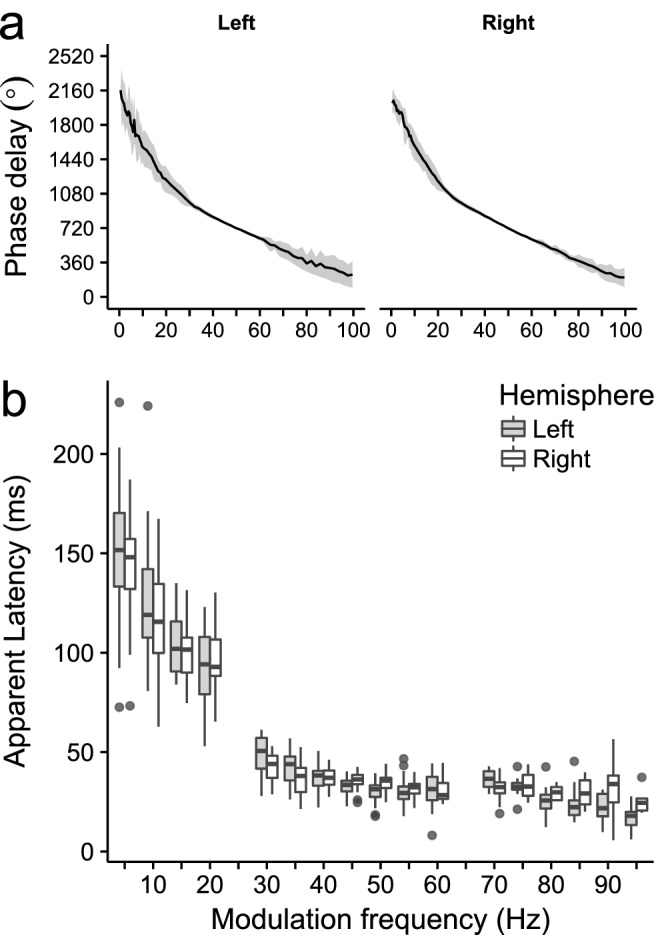
Phase delay and apparent latency. (**a**) Group average phase delays ± one standard deviation as a function of modulation frequency for the left and right hemispheric channel . The phase delay at 50 Hz of each individual was set to 720° for illustrative purposes, note that this affects the presented standard deviations. (**b**) the apparent latencies as a function of modulation frequency. It is clear from (**b**) that with the increase of modulation frequency the predominate generators of the ASSR shift downwards in the ascending auditory pathway.

Phase delays and the derived apparent latencies were used to gain insight in the generators from which the ASSRs to the different modulation frequencies originate. Phase delays within the 0.5–100 Hz region decreased with increasing modulation frequency (Fig. 9a). The apparent latencies as derived from the slopes of the phase delays showed that there are three main clusters of latencies, namely from 0.5 to 25 Hz, 25–65, and 65–100 Hz (Fig. 9b). The average apparent latencies as derived from these regions were 118.2 (SD = 32.2), 35.2 (SD = 8.1), and 24.9 (SD = 9.2) ms. There was a significant effect of modulation frequency on the apparent latencies for both the left (*H*(16) = 222.98, *p* < 0.001) and right hemispheric channel (*H*(16) = 204.27, *p* < 0.001). There was a significant decreasing effect on the apparent latency with increasing modulation frequency for both the left (*J* = 5214, p < 0.001) and right (*J* = 7630, p < 0.001) hemispheric channel. This indicates that the activity of the generators responsible for the ASSRs shift up in the auditory pathway with decreasing modulation frequency. A two-way ANOVA was used to gain insight in the effect of the modulation frequency and hemispheric channel on the apparent latency for ASSRs evoked for modulation frequencies from predominantly brainstem regions (i.e. modulation frequencies > 70 Hz). There was a significant main effect of modulation frequency (*F*(5) = 9.585, *p* < 0.001) and hemispheric channel (*F*(1) = 8.585, p < 0.004) and there was also a significant interaction between the two (*F*(5) = 2.472, p < 0.036). Post-hoc testing and after correction for multiple comparison did, however, not result in a significant difference between the response amplitudes measured with the left and right hemispheric channels for ASSRs to modulation frequencies between 70 and 100 Hz. ASSRs evoked within the 90–100 Hz region and recorded with the left hemispheric channel resulted in the lowest average latency, namely 16.7 ms (SD: 5.9 ms).

## Discussion

The goal of the present study was to assess the stimulus-evoked phase-locked activity to a broad range of modulation frequencies along the auditory pathway (i.e. the TMTF_ASSR_) and its inter-subject variability. Our study is the first, as far as the authors are aware, that assesses the TMTF_ASSR_ with a high frequency resolution and long recording times, enabling a thorough and individual assessment of the characteristics of phase-locked activity across the auditory pathway.

We found, in addition to the general TMTF_ASSR_ which has peaks of phase-locked activity around 30–60 Hz and 80–100 Hz^13,28,38^, that the TMTF_ASSR_ is characterized by a large intersubject variability. This is characteristic for the phase-locked activity that originates from the cortical regions^16–18,54^ and is elicited with modulation frequencies ≤ 20 Hz. Although most subjects had limited phase-locked activity within the theta range, the TMTF_ASSR_ ≤ 20 Hz shows distinct and subject specific patterns of phase-locked activity which are robust over time, and are therefore considered to be inherent to each subject. In literature, phase-locked amplitude functions ≤ 20 Hz have been characterized by a general decrease of amplitude as a function of increasing modulation frequency^40,41,55^. However, these studies only use a limited number of modulation frequencies to assess the TMTF_ASSR_ and report group averages. Our results show that at a group level this amplitude decrease as a function of increasing modulation frequency is indeed present and that there are peaks of activity at ~ 10 and ~ 20 Hz^20^, but this general pattern is the result of large inherent intersubject variability. The average latency of ASSRs evoked with modulation frequencies ≤ 20 Hz was 118.2 ms, indicating a cortical contribution to these responses. These latencies are in line with those reported in other studies^20,40^. In addition, our data show that there is an increase in latency of ~ 50 ms when the average response frequency decreases from 20 to 5 Hz. In contrast to ASSRs evoked with modulation frequencies > 20 Hz, these response amplitudes were not correlated across all modulation frequencies within this range, especially < 10 Hz, suggesting that the responsiveness of these cortical regions to specific modulation frequencies differ across subjects and potentially reflect unique processing characteristics of envelope modulated stimuli. These results also indicate that investigating just a few specific modulation frequencies ≤ 20 Hz to study specific effects of phase-locking and auditory functioning is potentially not representative of the overall phase-locked activity within the specific region under investigation.

Correlation analysis revealed that the 2.5 Hz ASSR amplitude was positively correlated with ASSR amplitudes within the ~ 50–100 Hz range, Pearson’s *r* varied from 0.44 to 0.86. One explanation is that phase-locked activity in the low delta and gamma band originates (at least partly) from the same generator which has a similar responsiveness to modulation frequencies within this range. An alternative hypothesis is a possible interaction between the activity of the two generators from which the delta and gamma ASSRs originate. Phase-locked activity of endogenous oscillations in the delta/theta bands has been postulated to modulate the activity in the gamma band for the processing of auditory stimuli^56,57^.

ASSRs evoked with modulation frequencies within the 30–60 Hz regions are the most reported in the literature^13,28,38^. We found that the f_peak_ (i.e. the modulation frequency that evokes the highest response within this region) across subjects spanned a range of 10 Hz, namely from 40 to 50 Hz. This variability is consistent with previous reports^38,58,59^. Zaehle et al.^59^ relate this subject specific f_peak_ to the resonance frequency of the auditory pathway. Furthermore, Poulsen et al.^38^ reported a positive correlation with the f_peak_ and increasing age, and Baltus and Herrmann^58^ found that subjects with higher peak frequencies also performed better in a gap detection task. Although f_peak_ within the 30–60 Hz region differed across subjects, the phase-locked activity within this region is highly correlated. In addition, the apparent latency in this range was relatively stable and was, on average, 35.2 ms, which is similar to those reported in the literature^44,60,61^. This indicates that the scalp recorded ASSRs within this range originate from the same generators, and although peak frequencies differ across subjects, the choice of modulation frequency presumably does not have a large effect on relative measures that assess within subject effects such as the relation between the ASSR and loudness^30,31^ or differences in modulation detection across different tonotopical regions^27,29^. Nevertheless, one should take this into account when comparing response amplitudes across populations, because a significant difference in activity at a specific modulation frequency could also indicate a shift in f_peak_.

ASSRs evoked with modulation frequencies ranging from 70 to 100 Hz shared the same overall amplitude characteristics, predominately for the left hemispheric channel, as is apparent from the correlation analysis. This left hemispheric lateralization was also apparent from our lateralization analysis. Hemispheric lateralization for ASSR evoked with these higher modulation frequencies, as recorded with EEG, needs to be interpreted with caution. Given that these responses predominantly originate from brain stem structures^15^ indicates that the dipole originating from these sources is best recorded with an ipsilateral configuration. This ipsilateral configuration is even more apparent from the results of Poelmans et al.^23^, who found that the lateralization of the 80 Hz ASSR depends on the stimulated ear. Although, post-hoc analysis did not yield significant effects, it is of interest that the lowest latency (16.7 ms) was found for the left hemispheric channel and for ASSRs in the 90–100 Hz range. This suggest that ASSRs recorded with the left hemispheric channel capture a larger contribution from generators located at earlier stages of the auditory pathway compared to other modulation frequencies. Purcell and John^62^ found, similar as our results, that the intersubject variability in response patterns for ASSRs evoked within the 70–100 Hz region can be rather large. These intersubject differences, especially for ASSR in the lower end of this range (i.e., 70–80 Hz) can originate from a different responsiveness of the brain stem generator(s) across subjects, but could also originate from a different combination, and dipole orientation of the generators that contribute to the scalp potential^53^.

## Conclusion

We assessed the stimulus evoked phase-locked activity to modulations ranging from 0.5 to 100 Hz (TMTF_ASSR_) in the normal-hearing young adult human auditory pathway. We found that the group averages showed a low-pass pattern with the exception of peaks of activity at 10, 20, 40–50 and 80–100 Hz. However, at an individual subject level we found a remarkable variability in response patterns, especially for the phase-locked activity originating from predominantly the cortical structures of the auditory pathway (i.e. activity to modulation frequencies ≤ 20 Hz). These results show that ASSRs to single modulation frequencies do not reflect the responsiveness of a specific frequency region and that multiple modulation frequencies are needed to gain full insight in the phase-locked activity of the cortical regions to modulation frequencies ≤ 20 Hz. Furthermore, we found that the cortical response patterns were highly robust over time indicating that the phase-locked activity, to modulation frequencies present in the speech envelope, is inherent to each subject. In addition, we also found that the peak with the most activity within the 40 to 50 Hz range differed across subjects. This indicates that just using a single frequency to assess the phase-locked activity within this frequency region potentially does not correctly reflect the maximum activity within the region of interest. The results of the present study demonstrate the importance of individual variability when assessing phase-locking in the auditory pathway and will aid future research designs with the selection of modulation frequencies to assess the relation between the evoked phase-locked activity and functional outcomes, or to assess specific generators located at different regions along the auditory pathway.
